# Antimicrobiota vaccine induces lysine-mediated modulation of tick immunity affecting *Borrelia* colonization

**DOI:** 10.1093/femsec/fiaf082

**Published:** 2025-08-14

**Authors:** Lourdes Mateos-Hernandez, Lianet Abuin-Denis, Alejandra Wu-Chuang, Apolline Maitre, Helena Roháčková, Ryan O M Rego, Elianne Piloto-Sardiñas, James Valdes, Stefania Porcelli, Aurelie Heckmann, Sara Moutailler, Covadonga Lucas-Torres, Martin Moos, Stanislav Opekar, Myriam Kratou, Dasiel Obregon, Alejandro Cabezas-Cruz

**Affiliations:** ANSES, INRAE, Ecole Nationale Vétérinaire d'Alfort, UMR BIPAR, Laboratoire de Santé Animale, Maisons-Alfort F-94700, France; ANSES, INRAE, Ecole Nationale Vétérinaire d'Alfort, UMR BIPAR, Laboratoire de Santé Animale, Maisons-Alfort F-94700, France; Animal Biotechnology Department, Center for Genetic Engineering and Biotechnology, Avenue 31 between 158 and 190, PO Box 6162, Havana 10600, Cuba; ANSES, INRAE, Ecole Nationale Vétérinaire d'Alfort, UMR BIPAR, Laboratoire de Santé Animale, Maisons-Alfort F-94700, France; ANSES, INRAE, Ecole Nationale Vétérinaire d'Alfort, UMR BIPAR, Laboratoire de Santé Animale, Maisons-Alfort F-94700, France; Institute of Parasitology, Biology Centre, Czech Academdiseases by targeting metabolite pathways.y of Sciences, Ceske Budejovice, Czech Republic; Faculty of Science, University of South Bohemia, Ceske Budejovice, Czech Republic; Institute of Parasitology, Biology Centre, Czech Academdiseases by targeting metabolite pathways.y of Sciences, Ceske Budejovice, Czech Republic; Faculty of Science, University of South Bohemia, Ceske Budejovice, Czech Republic; ANSES, INRAE, Ecole Nationale Vétérinaire d'Alfort, UMR BIPAR, Laboratoire de Santé Animale, Maisons-Alfort F-94700, France; Direction of Animal Health, National Center for Animal and Plant Health, Carretera de Tapaste y Autopista Nacional, Apartado Postal 10, 32700 San José de las Lajas, Mayabeque, Cuba; Institute of Parasitology, Biology Centre, Czech Academdiseases by targeting metabolite pathways.y of Sciences, Ceske Budejovice, Czech Republic; Centre Algatech, Institute of Microbiology, Czech Academy of Sciences, Novohradská 237, Třeboň 37901, Czech Republic; ANSES, INRAE, Ecole Nationale Vétérinaire d'Alfort, UMR BIPAR, Laboratoire de Santé Animale, Maisons-Alfort F-94700, France; ANSES, INRAE, Ecole Nationale Vétérinaire d'Alfort, UMR BIPAR, Laboratoire de Santé Animale, Maisons-Alfort F-94700, France; ANSES, INRAE, Ecole Nationale Vétérinaire d'Alfort, UMR BIPAR, Laboratoire de Santé Animale, Maisons-Alfort F-94700, France; Laboratoire de Chimie Moléculaire (LCM), UMR 9168, CNRS, Ecole Polytechnique, Route de Saclay, Palaiseau 91120, France; Institute of Parasitology, Biology Centre, Czech Academdiseases by targeting metabolite pathways.y of Sciences, Ceske Budejovice, Czech Republic; Laboratory of Analytical Biochemistry and Metabolomics, Biology Centre, Czech Academy of Sciences,České Budějovice 37005, Czech Republic; Laboratory of Analytical Biochemistry and Metabolomics, Biology Centre, Czech Academy of Sciences,České Budějovice 37005, Czech Republic; Laboratory of Microbiology, National School of Veterinary Medicine of Sidi Thabet, University of Manouba, Manouba 2010, Tunisia; School of Environmental Sciences, University of Guelph,Guelph, ON N1H 2W1, Canada; ANSES, INRAE, Ecole Nationale Vétérinaire d'Alfort, UMR BIPAR, Laboratoire de Santé Animale, Maisons-Alfort F-94700, France

**Keywords:** tick microbiota, *Borrelia*, metabolites, lysine, vector competence, tick physiology, tick immunity, defensins

## Abstract

Tick microbiota influences *Borrelia* colonization, but changes in the microbiota-derived metabolite and how this affects tick physiology and vector competence is unclear. We investigated whether microbiota-induced metabolite modifications influence tick physiology and pathogen transmission. Using an antimicrobiota vaccine (live *Escherichia coli*) to immunize mice, we generated host antibodies that modulated the tick microbiome, decreasing bacterial abundance and increasing lysine levels in ticks. Elevated lysine correlated with increased tick weight. Lysine supplementation experiments enhanced defensin expression with DefMT6 exhibiting anti-*Borrelia* activity, reducing pathogen load in ticks. Our findings demonstrate that antimicrobiota vaccines induce metabolite changes, affecting tick physiology, immunity, and vector competence. These insights open new avenues for developing microbiota-targeted strategies to control tick-borne diseases

## Teaser

A vaccine targeting tick microbiota increases lysine levels, boosting tick immunity, and reducing Borrelia colonization, offering a novel strategy for controlling tick-borne diseases.

## Introduction

Ticks are vectors of a wide range of pathogens that pose significant health risks to humans and animals worldwide (Sonenshine. 2013). Among these pathogens, *Borrelia afzelii*, one of the main infectious bacterial species in Europe that is the causative agent of Lyme borreliosis, is of particular concern (Madison-Antenucci et al. 2020, Strnad et al. 2023). Understanding the factors that influence tick–pathogen interactions is crucial for developing effective strategies to control tick-borne diseases. The tick microbiome has emerged as a critical modulator of both tick physiology and vector competence (Narasimhan et al. 2015). Alterations in the composition and function of the tick microbiota can significantly impact the ability of pathogens to colonize and persist within the tick vector (Hussain et al. 2022).

Recent studies have demonstrated that antimicrobiota vaccines targeting specific bacterial taxa within the tick microbiome can modulate tick-feeding behavior (Mateos-Hernández et al. 2020a) and alter the taxonomic and functional profiles of the microbial community (Mateos-Hernández et al. 2021). For instance, immunization of vertebrate hosts with live bacterial vaccines targeting keystone taxa such as *Escherichia*–*Shigella* induces the production of specific antibodies that, when ingested by feeding ticks, reduce the abundance of these bacteria in the tick microbiome and decrease overall species diversity (Mateos-Hernández et al. 2021). This targeted perturbation restructures the hierarchy of nodes in bacterial co-occurrence networks and reduces the resistance of the microbial network to taxa removal. Moreover, functional predictions indicate that such immunization reduces the abundance of pathways involved in lysine degradation within the tick microbiome (Mateos-Hernández et al. 2021), suggesting a potential increase in lysine levels in the tick midgut.

Furthermore, perturbation of the tick microbiota through antimicrobiota antibodies or the introduction of novel commensal bacteria has been shown to reduce the colonization of *B. afzelii* in *Ixodes ricinus* ticks (Wu-Chuan et al. 2023). The pathogen itself induces changes in the tick microbiota composition, beta diversity, and bacterial community assembly. Disrupting the pathogen-induced modulation of the microbiota leads to lower pathogen loads in ticks (Wu-Chuang et al. 2023), indicating that *Borrelia* is highly sensitive to microbiota perturbations.

Building upon these findings, a critical question arises: how does metabolite changes induced by microbiota perturbation influences tick physiology and vector competence? We hypothesize that the reduction of *Escherichia*-derived lysine degradation enzymes following antimicrobiota vaccination leads to increased lysine levels in the tick midgut. Here we investigated whether microbiota-induced metabolite modifications following antimicrobiota vaccination affects tick physiology, immunity, and vector competence. By immunizing mice with a live *Escherichia coli* vaccine, serving as an antimicrobiota vaccine, we generated host antibodies that modulate the tick microbiome when ticks feed on these mice, leading to increased lysine levels within the ticks.

Our findings reveal that antimicrobiota vaccines induce metabolite changes—specifically elevated lysine—, leading to alterations in tick physiology, immunity, and vector competence.

This study provides new insights into how ticks respond to microbiota perturbations, specifically through alterations in lysine metabolism, and highlights potential strategies for controlling tick-borne diseases by targeting metabolite pathways.

## Materials and methods

### Ethics statement

*In vivo* experiments were performed at the Animal Facility of the Laboratory for Animal Health of the French Agency for Food, Environmental and Occupational Health & Safety (ANSES), Maisons-Alfort, France, according to French and International Guiding Principles for Biomedical Research Involving Animals (2012). The procedures were reviewed and approved by the Ethics Committee (ComEth, Anses/ENVA/UPEC), with animal experimentation permit numbers, E 94 046 08 and APAFIS#31496–2021051213296446 v3.

### Animals and housing conditions

Six-week-old female C3H/HeN mice were purchased from Charles River and kept for adaptation for 1 week before conducting experiments. During the study, mice were maintained in green line ventilated racks at −20 Pa, with food and water ad libitum. The mice were kept at controlled room temperature (RT, 20°C–23°C) and a 12-h light: 12-h dark photoperiod regimen. The number of mice per cage was limited to five. Animals were monitored twice a day by experienced technicians and deviations from normal behaviors or signs of health deterioration were recorded and reported.

### Tick maintenance

*Ixodes ricinus* nymphs used in these experiments were obtained from our pathogen-free colony maintained at the tick rearing facility of ANSES (The French Agency for Food, Environmental and Occupational Health & Safety, Maisons-Alfort, France; with animal experimentation permit number APAFIS #35511–2022022111197802 v2) and reared at 22°C with 95% relative humidity and a 12 h light/dark cycle.

### Live bacteria immunization

Live bacteria vaccine was prepared using *E. coli* BL21 (DE3, Invitrogen, Carlsbad, CA) as previously described (Mateos-Hernández et al. 2020a). Briefly, *E. coli* culture was washed with phosphate buffer saline (PBS) 10 mM NaH2PO4, 2.68 mM KCl, 140 mM NaCl, pH 7.2 (Thermo Scientific, Waltham, MA), resuspended at 3.6 × 10^4^ colony-forming unit (CFU)/ml, and homogenized using a glass homogenizer. C3H/HeN mice were immunized subcutaneously with 100 µl of *E. coli* BL21 (1 × 10^6^ CFU per mouse) in a water-in-oil emulsion containing 70% Montanide™ ISA 71 VG adjuvant (Seppic, Paris, France), with a booster dose 2 weeks after the first dose. Control mice received a mock vaccine containing PBS and adjuvant.

### Tick infestation

Before tick infestation, mice were anesthetized by isoflurane and a 2-cm-outer-diameter Ethylene-Vinyl Acetate (EVA)-foam capsule was glued on their shaved backs using a nonirritating latex glue as described in previous work (Mateos-Hernandez et al. 2020b). Each mouse in the different groups with Mock vaccine or Live Bacteria was infested with 20 *I. ricinus* nymphs at day 42 (Fig. 3H). Ticks, previously placed in a syringe, were deposited to the capsule by slowly pushing the plunger, and then, a plastic lid was used to close the capsule (Mateos-Hernández et al. 2020b). Tick feeding was visually monitored twice a day. Engorged nymphs were weighed and collected in sterile tubes with holes and maintained with a light–dark (12 h/12 h) cycle in an incubator with >97% relative humidity at 22°C.

### Sera collection and preparation

Blood samples were collected at the beginning of the immunization protocol, and 1 week after the treatment for all groups. on sterile tubes without anticoagulant. For serum separation, the blood samples were incubated for 2 h at RT, allowing for clotting, and then centrifuged at 5000 × *g* for 5 min at RT, twice.

### Indirect Enzyme-Linked Immunosorbent Assay (ELISA)

The levels of Abs reactive against bacterial proteins were measured in mice sera as previously reported (Mateos-Hernández et al. 2020a). The 96-well ELISA plates (Thermo Scientific, Waltham, MA) were coated with 100 µl per well of 0.5 µg/ml of *E. coli* proteins and incubated for 2 h at RT with gentle continual shaking at 100 r/m. Subsequently, plates were incubated overnight at 4°C. The antigens were diluted in carbonate/bicarbonate buffer (0.05 M, pH 9.6). The next day, wells were washed three times with 100 µl of PBS containing 0.05% (vol/vol) Tween 20, and then blocked by adding 100 µl of 1% bovine serum albumin (BSA)/PBS for 1 h at RT and gentle continual shaking at 100 r/m. After three washes, sera samples, diluted 1:100 in 1.5% BSA/PBS, were added to the wells and incubated for 1,5 h at RT at 100 r/m. The plates were washed three times and 100 µl per well of horseradish peroxidase (HRP)-conjugated Abs (goat anti-mouse IgG and IgM) (Sigma-Aldrich, St. Louis, MO) was added at 1:1500 dilution in PBS and incubated for 1 h at RT at 100 r/m. The plates were washed three times and the reaction was developed with 100 µl ready-to-use TMB solution (Promega, Madison, WI) at RT for 20 min in the dark, and then stopped with 50 µl of 0.5 M H2SO4. Optimal antigen concentration and dilutions of sera and conjugate were defined using a titration assay. The optical density (OD) was measured at 450 nm using an ELISA plate reader (Filter-Max F5, Molecular Devices, San Jose, CA). All samples were tested in triplicate, and the average value of three blanks (no Abs) was subtracted from the reads.

### Tick's metabolites extraction protocol

Pools of ticks of ~35.0 mg (three pools per condition live vaccine and mock vaccine); were collected and polar metabolites were extracted following the protocol described by Beckonert et al. (2007). Briefly, after the feeding process, ticks were recovered in a 2 ml Eppendorf tube with a mixture of methanol/water CH_3_OH/H_2_O (400 µl/65 µl) and the metabolism quenched at −20°C. Pools were ground using a ball TissueLyser device (Quiqgen, Hilden, Germany). Supernatants were then recovered, 200 µl of chloroform (CHCl_3_) was added, and the tubes were vortexed. Then, CHCl_3_ and H_2_O (200 µl each) were added, and the tubes were vortexed again. The tubes were placed at 4°C for at least 15 min to accelerate the extraction, then centrifuged for 15 min. Aqueous phases were isolated and the solvent was evaporated under N_2_ gas (XcelVap), and finally the residual water was eliminated using a centrifugal vacuum concentrator device (SpeedVac SC110, Savant).

### NMR sample preparation

Dried samples were recovered in 500 µl of deuterated buffer (PB), prepared with 1 mM Trimethylsilylpropanoic Acid-d4, sodium salt (TSP) as internal reference and 6 mM sodium azide (NaN_3)_ to avoid enzymatic reactions that can degrade the metabolites in the sample. The homogenized solution was then placed in a 5 mm NMR tube. NMR experiments were recorded on a 400 MHz Bruker Avance I with a QNP 5 mm probe and a SampleXPress Lite changer operating at RT. The operating software was Topspin 2.1. ^1^H 1D NOESY-*presat* spectra were acquired for all the samples with a total time of 35 min each.

### Metabolomics analysis

Spectra were calibrated, phase-adjusted, and baseline-corrected with Topspin 4.3 (Bruker Biospin). Metabolic identification was possible thanks to Chenomx NMR Suite (version 10.1, Chenomx Inc., Edmonton, Canada). NMRProcFlow software (http://nmrprocflow.org/, version 1.4) was used for the spectral binning (variable size bucketing) after normalization to the total spectral area. Data matrices consisting of 81 variables (bins) and 6 samples were then analyzed with SIMCA 18 (version 18.0, Umetrics, Sweden) for multivariate data analysis, and with Metaboanalyst (http://www.metaboanalyst.ca/; version 6.0) for univariate data analysis.

Volcano plot was constructed using the data matrix generated after data normalization and spectral binning. The analysis was performed using Metaboanalyst 6.0 (http://www.metaboanalyst.ca/), without any further data transformation or scaling. Statistical difference between control and vaccine groups was based on selected raw *P*-values (<.1) and FC (higher or lower than 1).

### Metabolite extraction for mass spectrometry and sample preparation

Tick pools were prepared for analysis, consisting of groups of three ticks each: three pools from unfed ticks, two pools from fully engorged ticks, and one pool of blood collected from the animals on which the ticks had fed. Acidic methanol (0.1 M HCl in methanol) was added to each sample at a ratio of one part sample to five parts acidic methanol (e.g. 10 mg or 10 ml of sample combined with 50 ml of acidic methanol). To enhance metabolite extraction, the ticks were subsequently crushed with acidic methanol (0.1 M HCl in methanol) was added to the sample at a ratio of one part sample to five parts acidic methanol (e.g. 10 μl or mg of sample with 50 μl of acidic methanol). Ticks were then crushed to facilitate metabolite extraction.

The measurement of lysine was performed using a slightly modified ethyl chloroformate (ECF) method (Opekar et al. 2021).

Tick extract samples were stored in 400 µl (MeOH:0.1 M HCl (aq) = 95:5 v/v). Samples were centrifuged (4°C/7 000 r/m/10 min) and 200 µl of the supernatant was dried in a culture test tube in a speedvac.

Mouse blood (200 µl) was precipitated directly with 800 µl (ACN:MeOH 1:1 v/v) and centrifuged (4°C/7 000 r/m/10 min). A total of 200 µl of the supernatant was dried in a culture test tube in the speedvac.

The procedure was continued with the addition of internal standards Lys-13C6 and Trp-13C11, 10 µl, (0.1 nmol/µl each) to the dried extract. A solution of 0.1 M NaOH:EtOH 6:1 v/v (90 µl) was added and vortexed. Subsequently, 40 ul CHCl_3_:ECF 7:1 v/v was added. The mixture was briefly vortexed and allowed to stand for 1–2 min. A 40 μl mixture of ethanol:pyridine 2:1 was added, followed by a 40 μL CHCl_3_:ECF (7:1 v/v) addition. Further, 40 μl of 1 M NaOH aqueous solution was added and the third addition of 40 μl CHCl_3_:ECF (7:1, v/v) was performed. A 100 μl portion of the lower CHCl_3_ layer was withdrawn and carefully evaporated under nitrogen and redissolved in 50 μL 30% MeOH prior to LC–MS analysis.

LC–HRMS analysis was performed on a Dionex Ultimate 3000 liquid chromatograph coupled to a high-resolution mass spectrometer Q Exactive Plus (both Thermo Fisher Scientific, San Jose, CA). A 150 × 3 mm, 2.6 μm Kinetex C18 column (Phenomenex, Torrance, USA) was used for separation of the detected components at 35°C using methanol and water containing 5 mmol l^−1^ ammonium formate (pH = 4.5) as the mobile phase. The initial methanol concentration of 30 vol% was linearly increased to 100% within 10 min and the composition kept at 100% for 1 min; flow rate was set 400 μl min^−1^; injection volume, 5 μl. Full scan HRMS positive ion ESI spectra were recorded in a mass range 85–750 daltons at 70 000 resolution (m/z 200). The Q-Exactive settings were: scan rate at ±3 Hz, 3 × 106 automatic gain control (AGC) target and maximum ion injection time 200 ms; ion source parameters 3 kV spray voltage, 350°C capillary temperature, sheath gas at 40 au, aux gas at 10 au, spare gas at 1 au, probe temperature 350°C and S-Lens level at 60 au. For accurate the mass measurements, lock masses m/z 100.07569, 149.02332.0, 279.15909, and 391.28428 were used. For the data processing Excalibur software 4.1.0.0 was used.

### Quantification of lysine

The levels of lysine in tick were measures using two different methods. Tick nymphs were collected in pools of three at three different stages: unfed, after 2 days of feeding, and fully engorged, from both control and immunized mice. The samples were placed in sterile tubes, and metabolites were extracted following the manufacturer's instructions for either the Quantitative Lysine Assay or mass spectrometry quantification. Lysine levels were measured using the Quantitative Lysine Assay, adhering to the manufacturer's instructions or metabolite's extraction protocol for mass spectrometry.

### Artificial feeding

Artificial feeding technique was previously described by Bonnet et al. (Bonnet et al. 2007). Briefly, *I. ricinus* nymphs from the colonies of UMR BIPAR, Maisons-Alfort, France, were placed in two tick chambers (*n* = 40). A 10 cm diameter gerbil skin was sterilized in 70% alcohol, rinsed twice with sterile distilled water and twice with sterile PBS 1X, and sterilized again in a solution containing 2.5 ml (10 mg/ml) gentamicin, 500 μl (250 μg/ml) amphotericin B, 2.5 ml (50 mg/ml) streptomycin, and 2.5 ml (50 U/ml) penicillin (Sigma-Aldrich, St. Louis, MO). The skin was placed between the tick chamber and the glass feeder. Defibrinated sheep blood (Atlantis, France) was decomplemented 30 min at 56°C. A total of 20 μl Amphotericin B (250 µg/ml, Invitrogen, Germany), 100 μl gentamycin (10 mg/ml, Invitrogen, Germany) and 34 μl heparin (10 KU/ml, Merck, Germany) were added to the blood before use. Each feeder was filed with 3.5 ml of blood, heated at 37°C and was changed twice a day for 14 days. The two artificial feeding system contained (i) 20 µM of lysine diluted in 70 µl of PBS and (ii) 35 µl of sterile PBS as a control. Twice per day nymphs were observed, collected and weighted if the feeding was completed and stored at −80°C.

### Bacterial culture

Low passage *B. afzelii* CB43 were started from glycerol stocks and grown in Barbour–Stoenner–Kelly (BSK)-H (Sigma-Aldrich, St. Louis, MO) media containing 6% rabbit serum and were kept at 33°C for 7 days.

### Complement and serum bactericidal activity assay

A baby rabbit complement (BRC, Bio-Rad) solution (final concentration of 2.5%; v:v) was mixed with an *E. coli* suspension, diluted 200 000 times. Sera samples of each mouse group (Mock, or *E. coli* vaccine groups) were pooled and incubated at 56°C for 30 min. For each group, the heat-inactivated sera were mixed to the suspension of *E. coli* and BRC (with a final concentration of 5%). A control group containing only *E. coli* and BRC was used as the growth reference. The bacterial suspensions were incubated at 37°C and aliquots collected after 1, 2, 3, and 4 h were spread on Tryptic Soy Agar (Sigma-Aldrich, St. Louis, MO). After an overnight incubation of the plates at 37°C, the number of CFUs was recorded.

### DefMT6 borreliacidal assay

To evaluate the effect of the Def MT6 peptide on *Borrelia*, 2 × 10^3^  *Borrelia* were incubated in duplicate with three different concentrations (5 µM, 10 µM, and 20 µM). Total volume was 100 µl. Control group was *Borrelia* culture incubated with 20 µM of a scrambled peptide. After 24 h of incubation at 34°C, 50 µl from each sample was transferred to a larger volume of fresh media to support further growth. The remaining 50 µl was transferred to fresh media after 48 h. *Borrelia* quantification was performed using a Petroff–Hausser counting chamber to assess differences in bacterial growth across conditions.

### Tick capillary feeding

Two capillary feeding experiments were conducted on unfed nymphs (*n* = 80). Capillaries (3.5″; Drummond Scientific, Broomall, PA) were prepared by heating and pulling 1-mm glass capillary tubes using a glass micropipette puller (P-1000, Sutter Instrument, Novato, CA).

The nymphs were divided into DefMT6 and Scramble groups, with or without *B. afzelii* exposure. Ticks in the *Borrelia* groups were fed a mixture of *B. afzelii* culture (5 × 10^6^ spirochetes/ml, BSK-II media) and either DefMT6 or Scramble peptides, while ticks in the non-*Borrelia* groups were fed only the respective peptides. Ticks were maintained at 37°C and high humidity during the 3-h feeding period. Immediately afterward, ticks from the non-*Borrelia* groups were frozen for 16S analysis. In the *Borrelia* groups, half of the ticks were frozen after the 3-h feeding period, while the remaining half was kept under controlled humidity and light conditions for 24 h before being frozen for further *Borrelia* quantification.

### DNA extraction

Genomic DNA was extracted from fully engorged nymphs. DNA from individual nymphs were extracted at the end of the period of incubation from the capillary feeding experiment. Individual ticks were crushed with disposable probe. Genomic DNA was extracted from tick using a Nucleospin tissue DNA extraction Kit (Macherey–Nagel, Hoerdt, France). Each DNA sample from ticks was eluted in 20 µl of sterile water. Genomic DNA quality (OD260/280 between 1.8 and 2.0) was measured with NanoDrop™ One (Termo Scientifc, Waltham, MA).

### Quantitative real-time for defensins genes, relative abundance of Enterobacteriaceae and *B. afzelii* quantification

For defensin quantification, RNA from nymphs fully fed on different groups of artificial feeding were extracted using Trizol reagent (Invitrogen), and reverse transcription was performed with Superscript III (Invitrogen). Quantitative reverse transcription PCR (qRT-PCR) was performed using SYBR premix Ex Taq (Roche) in three biological replicates in a LightCycler 480 II (Roche). The specificity of the qRT-PCR amplicons for *DefMT2, DefMT3, DefMT4, DefMT6*, and *DefMT7* was confirmed through melting curve analysis, ensuring accurate amplification without nonspecific products or primer dimers. The ribosomal protein S4 (*rsp4*) was used as a reference gene (Koci et al. 2013). Primers sequences are described on Table 1. The relative mRNA level was quantified using the ΔΔCt method and expressed as a fold difference (Livak et al. 2001). ΔΔCt values were calculated in Microsoft Excel and final graphs and two tailed t-test statistics were obtained using GraphPad Prism 5 (GraphPad Software, La Jolla, CA).

**Table 1.** Sequences of primers used in this study.

Gene	Forward primer	Reverse primer
*DefMT2*	ACGAACTTGTACATCACAGAG	CAGCTTCCAACGTCCTCCGC
*DefMT3*	GAAGGTCCTTGCTGTCTCGCT	ATGGGCAGTAATAACCAC
*DefMT4*	GCTGGTCTGATCACTACATCG	CAAAGCTCCTGCAGTGACGAT
*DefMT6*	AGCACTTCCTGCTCACAAGAA	TTGATGATTCCCGAGCAGTAA
*DefMT7*	TTCATTATTCACGGTGGCGCT	CTCTACATTGCTGGGAGCAGG
*rps4* (HK)	GGTGAAGAAGATTGTCAAGCA	TGAAGCCAGCAGGGTAGTTGG
Enterobacteriaceae	ATGGCTGTCGTCAGCTCGT	CCTACTTCTTTTGCAACCCACTC
*Borrelia* spp. 23S rRNA	GAGTCTTAAAAGGGCGATTTAGT	CTTCAGCCTGGCCATAAATAG
*Borrelia* spp. 23S rRNA (probe)	AGATGTGGTAGACCCGAAGCCGAGT	

DNA extracted from whole nymphal tick was used to detect Enterobacteriaceae and *B. afzelii* abundance. For Enterobactericeae, 16S gene was amplified using the primers described in Table 1 for 40 cycles at 98°C for 10 s, 55°C for 30 s, and 72°C for 1 min, with a final extension at 72°C for 3 min in an automated thermal cycler (Perkin-Elmer Cetus, Gouda, The Netherlands). For *B. afzelli* detection, DNA from ticks underwent a preamplification step to improve pathogen DNA detection. For that, total DNA was preamplified using the PreAmp Master Mix (Fluidigm, CA) according to the manufacturer's instructions. Primers targeting the gene 23S rRNA for *Borrelia* spp. (Table 1) were pooled by combining an equal volume of each primer for a final concentration of 200 nM. The reaction was performed in a final volume of 5 μl containing 1 μl Perfecta Preamp 5X, 1.25 μl pooled primer mix, 1.5 μl distilled water, and 1.25 μl DNA. The thermocycling program consisted of one cycle at 95°C for 2 min, 14 cycles at 95°C for 15 s and 4 min at 60°C. At the end of the cycling program, the reactions were diluted 1:2 in Milli-Q ultrapure water. Subsequently, a quantitative PCR (qPCR) was carried out using the same aforementioned primers and an additional 23S rRNA probe (Table 1) in a LightCycler 480 (Roche, Meylan, France). The reaction mixture contained 6 μl of FastStart universal probe master (Roche), 0.12 μl of 20 µM of primers 23S rRNA-F, 23S rRNA-R and TaqMan probe 23S rRNA-probe, 2 μl of preamplified DNA or cDNA sample, and Milli-Q ultrapure water up to 12 µl. The amplification program consisted of the following: 95°C for 5 min, 45 cycles of 95°C for 10 s, and 60°C for 15 min. The spirochetes burden in ticks was obtained by interpolation of the CT value in a standard curve of “number of spirochetes vs CT” and then was normalized by the quantity of DNA in each sample.

### Microbiota analysis

#### Illumina library preparation and sequencing of the 16SrRNA gene

At least 200 ng of fully engorged nymphs DNA at≥ 20 ng/μl concentration was sent for amplicon sequencing of the bacterial 16S rRNA gene, which was commissioned to Novogene Bioinformatics Technology Co. (London, UK). Libraries were prepared with NEBNext® Ultra™IIDNA Library Prep Kit (New England Biolabs, MA). A single lane of Illumina MiSeq system was used to generate 251-base paired-end reads from the V4 variable region of the 16S rRNA gene using barcoded universal primers (515F/806R) in samples from nymphs fed with DefMT6 (*n* = 5), and scramble (*n* = 5). (Bioproject No PJRNA1193515.) The raw 16S rRNA gene sequences obtained from tick samples were deposited at the SRA repository.

#### Controls, identification, and removal of contaminants

Two extraction reagent controls were set in which the different DNA extraction steps were performed using the same conditions as for the samples but using water as template. DNA amplification was then performed on the extraction control in the same conditions as for any other sample. Possible contaminating DNA in samples for 16S rRNA gene sequencing was statistically identified with “decontam” package using the “prevalence” method. The prevalence is defined as the presence or absence across sample and the method used compares the prevalence of each sequence feature in true samples to the prevalence in negative controls to identify contaminants. Then, contaminants were removed from the dataset before downstream microbiome analysis (Davis et al. 2018).

#### Analysis of 16S rRNA gene amplicon sequences

The analysis of 16S rRNA gene sequences was performed using QIIME 2 pipeline (v.2021.4). Using DADA2 software implemented in QIIME2, 16S rRNA gene sequences were first demultiplexed and then quality trimmed based on the average quality per base of the forward and reverse reads. The total length was trimmed at 240 in forward and reverse reads, respectively. Consequently, reads were merged, and chimeric variants were removed. The resulting representative sequences were taxonomically assigned using a pretrained naïve Bayes taxonomic classifier based on SILVA database version 132 and the 515F/806R primer set. The resulting taxonomic data tables were collapsed at the genus level and taxa with <10 total reads and present in <30% of samples of each dataset were removed. The taxonomic data tables were used for network analysis.

#### Construction of bacterial co–occurrence networks

Co-occurrence network analysis was performed using the Sparse Correlations for compositional data (SparCC) method (Friedman et al. 2012) implemented in R studio. Taxonomic data tables were used to calculate the correlation matrix. Correlation coefficients with magnitude > 0.75 or < − 0.75 were selected. Network visualization and calculation of topological features and taxa connectedness (i.e. number of nodes and edges, modularity, network diameter, average degree, weighted degree, clustering coefficient, and centrality metrics) were performed using the software Gephi 0.9.2 (Bastian et al. 2009).

### Statistical analysis

Alpha and beta diversity of bacterial taxa were carried out on rarifed ASV tables. The alpha diversity was explored using the observed features (Bolyen et al. 2019), Faith's phylogenetic diversity (Faith et al. 1992) Shannon entropy (Shannon 1948), and Pielou's evenness index (Pielou et al. 1966) metrics. Differences in alpha diversity metrics between groups were tested with the Kruskal–Wallis test (*P* ≤ .05) using QIIME 2 (Bolyen et al. 2019). Beta diversity was explored using the Jaccard similarity coefficient of similarity using Vegan (Oksanen et al. 2021), Bray–Curtis dissimilarity index (Bray et al. 1957), unweighted and weighted Unifrac measures and compared between the groups using a permutational multivariate analysis of variance (PERMANOVA) test (*P* ≤ .01) on QIIME 2. The number of shared or unique nodes in the microbial networks of ticks from different experimental groups was visualized using Venn diagrams implemented in the online tool http://bioinformatics.psb.ugent.be/webtools/Venn/. Differences in relative Ab levels (i.e. OD) and number of *E. coli* colonies between groups of immunized mice in the different time points were compared using two-way ANOVA with Sidak's multiple comparison tests applied for individual comparisons. The unpaired nonparametric Mann–Whitney U test was used to compare the relative abundance of Enterobacteriaceae, quantity of lysine, tick weight, relative abundance of defensin genes, and the load of *B. afzelii* in ticks between different groups. Box plot for lysine bucket at 3.02 ppm obtained from univariate data analysis. The box plot is obtained from volcano plot (data not shown) representing the results from a *t*-test (*P*-value < .1) and fold-change analysis (different than 1). The Mann–Whitney U test and two-way analysis of variance (ANOVA) test followed by Sidak's multiple comparisons test were performed in the GraphPad 8 Prism software (GraphPad Software Inc., San Diego, CA). Differences were considered significant when *P* < .05.

## Results

### Microbiota perturbation driven by *E. coli*-specific antibodies induces lysine accumulation

Our previous research demonstrated that immunization with a live *E. coli* vaccine stimulates host antibodies that modulate the tick microbiome (Mateos-Hernández et al. 2020a, 2021). This modulation was associated with a relative reduction in the abundance of tick microbiota-derived enzymes *eutD* (EC.2.3.1.8) and *atoB* (EC.2.3.1.9) involved in lysine degradation (pathway P163–PWY) (Mateos-Hernández et al. 2021), ultimately contributing to a decreased capacity for pathogen colonization within the tick (Wu-Chuang et al. 2023).

**Figure 1.**
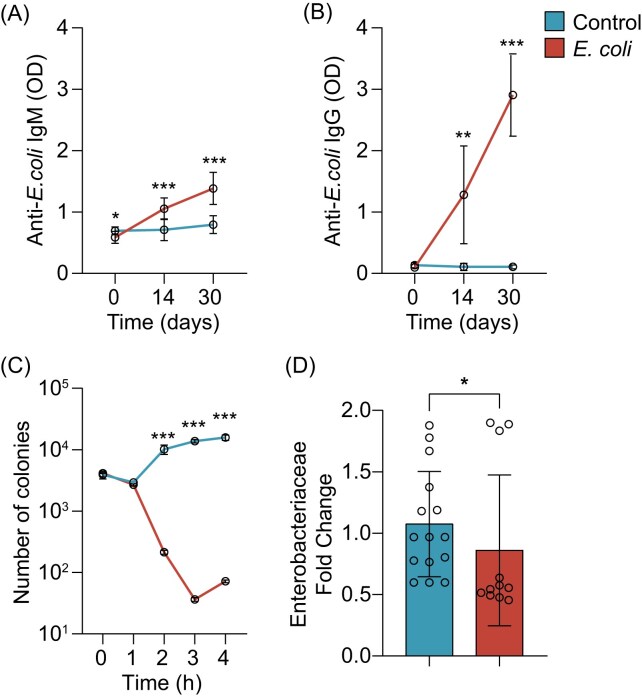
Microbiota perturbation induces bactericidal host antibodies. Levels of antibodies (A) IgM and (B) IgG specific to *E. coli* proteins were measured by semi-quantitative ELISA in sera of C3H mice immunized with live *E. coli* or a mock vaccine containing PBS (control) (*n* = 10). (C) Quantification of CFUs of *E. coli* over time in a complement-mediated bactericidal assay using sera from *E. coli* immunized and control mice (*n* = 5). (D) Quantification of Enterobacteriaceae by qPCR in *I. ricinus* ticks fed on mock or *E. coli*-immunized mice. Quantitative data are shown as individual data points (*n* = 30). All values are means ± SD. (A)–(C) two-way ANOVA with Sidak's multiple comparison test and (D) nonparametric Mann–Whitney test. *P < .05, **P < .01, ***P < .001.

We hypothesized that the increase in available lysine after microbiota perturbation could have a role in the resistance of ticks to pathogen colonization. To prove that host antibodies can increase lysine concentration in ticks, we immunized mice with the live *E. coli* vaccine which successfully elicited anti-*E. coli* IgM (Fig. 1A) and IgG (Fig. 1B) antibodies. These antibodies effectively decreased bacterial growth evidenced by the significant reduction of the number of CFUs *in vitro* (Fig. 1C) showing the bactericidal effect of the anti-*E. coli* antibodies. Immunization of mice with live *E. coli* vaccine also decreased the relative abundance of Enterobacteriaceae in ticks fed on immunized mice (Fig. 1D) confirming the ability of anti-*E. coli* antibodies to target bacteria.

**Figure 2.**
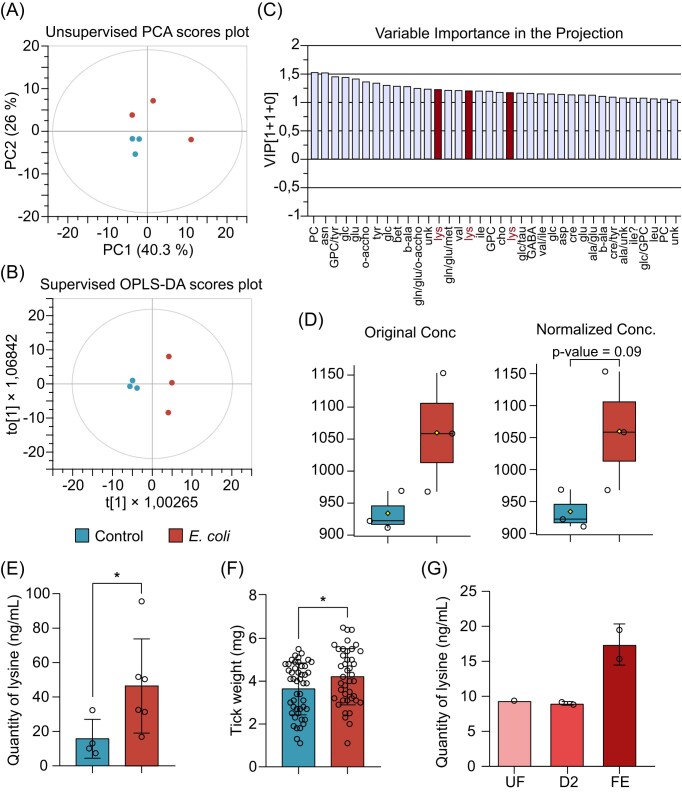
Microbiota perturbation induces expression of the metabolite lysine in ticks. Metabolomic analysis were performed in ticks fed on immunized and control mice. (A) Unsupervised PCA analysis of the 6 tick pool samples. The model is composed of three components (R^2^cum = 0.829, Q^2^cum = −0.0006). (B) Supervised OPLS–DA score plot of the 6 tick pool samples. The model is composed of 1 predictive component + 1 orthogonal component (R^2^Xcum = 0.629, R^2^Ycum = 0.991, Q^2^cum = 0.782, *P*-value = .3). (C) VIP plot from the OPLS–DA analysis exhibiting the variables contributing to the discrimination in model B. Horizontal axis represents the variables (bins) with a VIP value >1. Lysine bins are highlighted for clarification. (D) Box plot for lysine bucket at 3.02 ppm obtained from univariate data analysis. The box plot is obtained from volcano plot (data not shown) representing the results from a *t*-test (*P*-value < .1) and fold-change analysis (different than 1). (E) Quantification of lysine in *I. ricinus* ticks fed on mock or *E. coli*-immunized mice. (F) Weight of *I. ricinus* ticks fed on *E. coli*-immunized mice compared to ticks fed on mock-immunized mice. (G) Quantification of lysine in *I. ricinus* ticks during different days of tick engorgement on mice. UF: unfed; D2: 2 days post infestation; FE: Fully engorged ticks. Quantitative data are shown as individual data points. All values are means ± SD. (E) (*n* = 10) and (F) nonparametric Mann–Whitney test. *P < .05. (*n* = 80).

We next explored whether these host antibodies could trigger changes in the tick metabolome. We adopted an untargeted approach using metabolomic to identify metabolites whose abundance changed significantly in ticks fed on immunized mice compared to control ticks. An unsupervised principal component analysis (PCA) showed a separation of the control samples from the vaccine samples (Fig. 2A). The grouping of control samples confirms an effect of the vaccine.

To confirm this hypothesis, a supervised model was constructed. Both PLS–DA and OPLS–DA were envisioned, but OPLS–DA model was chosen to consider the orthogonal time information that we had from the samples (“S” for short, “L” for long time), together with their class (control or vaccine). OPLS–DA was assessed (Fig. 2B), showing a clear dispersion along the first predictive component between control and vaccine samples, suggesting the two conditions are significantly different. A slight scattering of the vaccine samples along the orthogonal component could be explained by the difference in time. The VIP plot (Fig. 2C) from the OPLS–DA shows that the three variables (bins) created for the amino acid lysine in our dataset exhibit a VIP > 1, indicating that these variables (bins) contribute to the sample discrimination. Therefore, univariate data analysis was run with the same dataset to assess the reliability of those three specific variables. From the volcano plot (Fig. S1, Table S1), only two of those three variables show a *P*-value < .1, one of them shown in Fig. 2D. From the depicted box plot, the significant variability of lysine between control and vaccinated samples is demonstrated, revealing that this amino acid was up regulated in the latter group. Metabolomics analysis results were further confirmed by Quantitative Lysine assay where significantly higher concentrations of lysine were found in ticks that fed on mice immunized with *E. coli* compared to ticks fed on control mice (Fig. 2E). Furthermore, we found that ticks fed on immunized mice had increased weight compared to the control ticks (Fig. 2F). Overall, these results show that immunization with a live *E. coli* vaccine can elicit bactericidal host antibodies which induce a decrease in bacterial abundance and an increase in available lysine in ticks, leading to a phenotypical change characterized by an increase in tick weight.

Endogenous lysine was consistently detected throughout the tick feeding process, with peak concentrations observed in fully engorged ticks, as confirmed by lysine quantification assays (Fig. 2G) and mass spectrometry (Table S2). This suggests that while *E. coli* immunization-induced microbiota perturbation increases lysine levels in ticks compared to controls, lysine concentration also naturally rises during feeding. These findings indicate a dual effect: the bactericidal activity of anti-*E. coli* antibodies reduces Enterobacteriaceae abundance, enhancing lysine availability, while feeding-driven lysine accumulation further supports tick physiology and may contribute to pathogen resistance.

### Artificial addition of lysine modulates defensin genes expression in *I. ricinus* ticks

**Figure 3.**
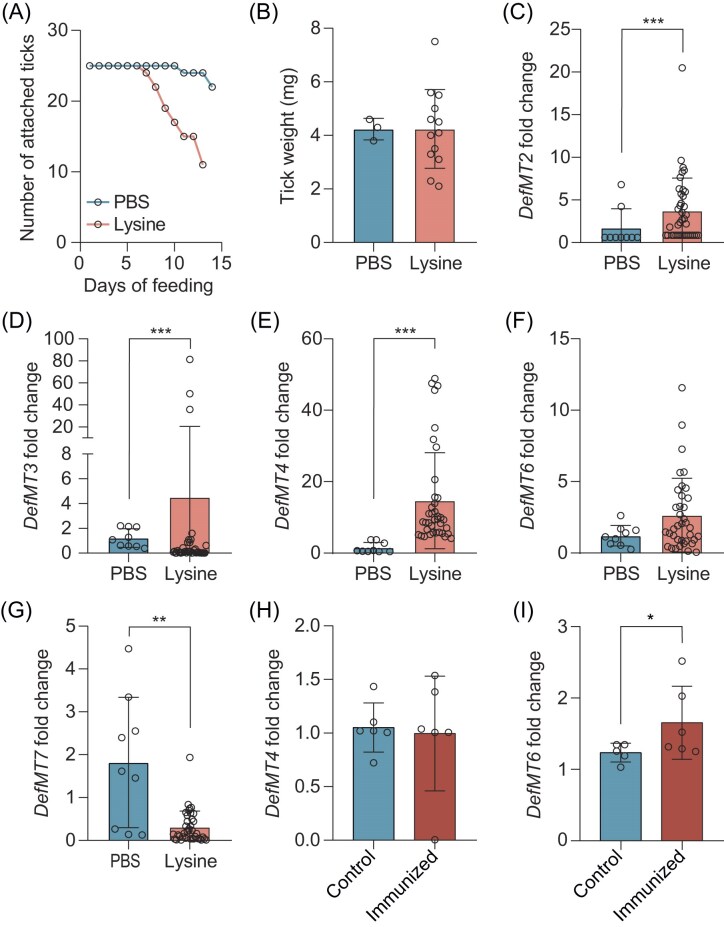
Lysine induces the expression of defensin genes in ticks. (A) Number of ticks attached to the membrane feeding system during different days of feeding on lysine-supplemented blood or PBS-supplemented blood (PBS). (B) Weight of ticks fed on blood supplemented with lysine compared to ticks fed on PBS-supplemented blood (PBS). Quantification of the expression of (C) defensin 2 (*DefMT2*), (D) defensin 3 (*DefMT3*), (E) defensin 4 (*DefMT4*), (F) defensin 6 (*DefMT6*), and (G) defensin 7 (*DefMT7*) by RT-qPCR in ticks fed on blood supplemented with lysine compared to control ticks. Quantification of the expression of (H) *DefMT4* and (I) *D efMT6* by RT-qPCR in ticks fed on mock (control) or *E. coli*-immunized mice (immunized). All values are means ± SD. Mann–Whitney test. *P < .05, **P < .01, ***P < .001. (*n* = 16).

To evaluate the role of lysine in the physiology of ticks, we allowed nymphs to feed artificially on blood supplemented with lysine. Lysine-fed nymphs showed faster feeding compared to the control group (Fig. 3A) but no significant changes in tick weight were observed between the different groups (Fig. 3B). We next wondered if the increase in lysine in ticks has an impact on the tick immune system. We therefore evaluated the expression of different defensins in ticks that received lysine. We found that the expression of *DefMT2, DefMT3*, and *DefMT4* was significantly higher in ticks fed with lysine-supplemented blood compared to the control group (Fig. 3C–E). Similarly, *DefMT6* expression showed an increasing trend in ticks fed with lysine (Fig. 3F). In contrast, *DefMT7* expression was lower in ticks fed with lysine compared to the control group (Fig. 3G).

As our previous findings indicated a significant increase in lysine concentration in ticks that fed on *E. coli*-immunized animals (Fig. 2E), we examined whether the expression of *DefMT4* and *DefMT6* was affected. We selected *DefMT4* because *DefMT3* and *DefMT4* had the highest expression levels in lysine-fed ticks and are isoforms, making *DefMT4* a representative of this group (Tonk et al. 2015). Although *DefMT6* only showed a slight increase in lysine-fed ticks, it was included due to its reported antimicrobial activity against *E. coli*. Quantification revealed no significant differences in *DefMT4* expression between ticks fed on mock- and *E. coli*-immunized groups (Fig. 3H). However, the expression of *DefMT6* was significantly higher in ticks fed on *E. coli*-immunized mice compared to those fed on mock-immunized mice (Fig. 3I). These findings suggest that lysine, induced by microbiota perturbation, can stimulate the production of antimicrobial peptides in ticks.

### *Ixodes ricinus* innate immune response regulates tick microbiota and tick borreliacidal activity

**Figure 4.**
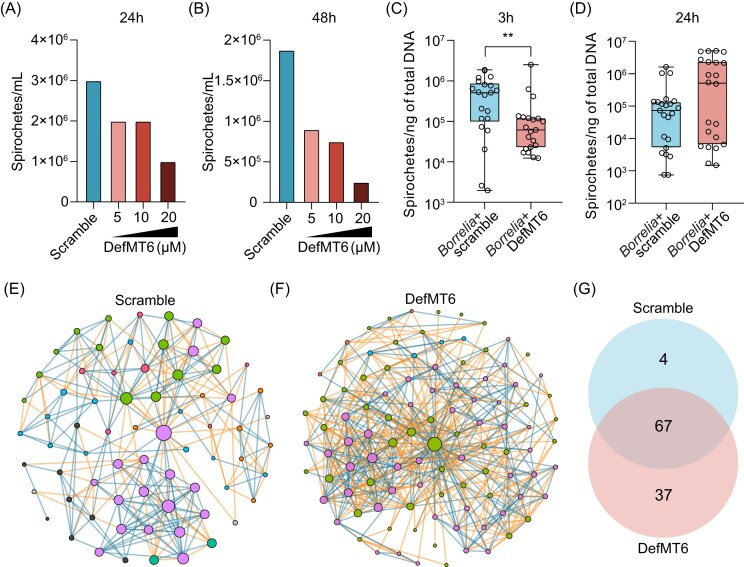
*Ixodes ricinus* innate immune response regulates tick borreliacidal activity. Quantification *in vitro* of the load of *B. afzelii* spirochetes after (A) 24 h and (B) 48 h of incubation of *Borrelia* with increased dose of defensin 6 (DefMT6). Quantification of the load of *B. afzelii* spirochetes at (C) 3 h and (D) 24 h in ticks capillary-fed with a solution containing *Borrelia* and defensin 6 (*Borrelia*+DefMT6) compared to control ticks capillary fed with the pathogen and a scramble (*Borrelia*+Scramble). Bacterial co-occurrence networks inferred from 16SrRNA gene sequences obtained from ticks treated with (E) scramble or (F) DefMT6 by capillary feeding. Nodes represent bacterial taxa and edges stand for a co-occurrence correlation (SparCC > 0.75 or < −0.75). Node size is proportional to the eigenvector centrality value and node color is based on the modularity class (nodes with the same color belong to the same cluster). Positive and negative interactions between co-occurring bacteria are represented by the dark blue and orange edges, respectively. Only nodes with at least one connecting edge are displayed. (G) Venn diagram showing the number of unique or shared nodes of bacterial co-occurrence networks inferred from the microbiota of ticks fed with scramble or DefMT6 by capillary feeding. Quantitative data are shown as individual data points. All values are means ± SD. (C), (D) Mann–Whitney test. **P < .01. (2 biological replicates, 80 ticks for capillary feeding, 40 for 16S rRNA sequencing).

We next assessed whether DefMT6 has an impact on pathogen survival. For that, *B. afzelii* was grown in culture media containing different concentrations of DefMT6 and compared to its growth in media with scrambled peptides. After 24 h of incubation, a decreasing trend of spirochete numbers was observed with increasing concentrations of DefMT6 (Fig. 4A). This reduction became more pronounced when *B. afzelii* spirochetes was quantified after 48 h of culture with DefMT6 (Fig. 4B).

To evaluate the impact of DefMT6 on *B. afzelii* colonization *in vivo*, we introduced DefMT6 in *Borrelia*-infected ticks using capillary feeding. After 3 h post-feeding, we found that the amount of *B. afzelii* colonizing the tick was significantly lower in ticks that received DefMT6 compared to control ticks fed with scrambled peptides (Fig. 4C). However, we found that this effect was lost after 24 h of incubation (Fig. 4D).

We next wanted to determine if DefMT6 has an impact on tick microbiome. Analysis of alpha diversity indexes (observed features, Shannon entropy, Pielou's evenness and Faith's phylogenetic diversity) showed that DefMT6 did not change the number of unique taxa, richness, evenness or the phylogenetic diversity on tick microbiome (Fig. S2A–D). Addition of DefMT6 to ticks did not result in a significantly change the beta diversity indexes (Jaccard, Bray-Curtis, Unweighted unifrac and Weighted unifrac) tested showing that DefMT6 did not impact the bacterial community composition and abundance of tick microbiota (Fig. S2E–H). However, we found that DefMT6 modulated drastically the bacterial community assembly (Fig. 4E, F) as evidenced by the increase in the number of nodes and edges (Table 2). Specifically, addition of DefMT6 led to an increase of nodes from 71 in the networks of the control condition to 104; the number of edges also showed an increase from 268 edges in the control group to 610 in the DefMT6 group (Table 2). Conversely, DefMT6 led to a decrease in the modularity, network diameter and weighted degree compared to the control group (Table 2). Comparison of the nodes identity showed that a high number of nodes (i.e. 67) were shared between the group that received DefMT6 and the control group (Fig. 4G, Table S3). We also found that microbial networks of ticks that received DefMT6 has more unique nodes (i.e. 37) compared to the control group (Fig. 4G). These results suggest that DefMT6 can modulate the tick bacterial assembly and impact pathogen survival within *I. ricinus* ticks.

**Table 2.** Topological features of microbial co-occurrence networks from noninfected ticks capillary-fed with DefMT6 or a scramble peptide.

Topological features	Experimental groups	
	Scramble	DefMT6
**Nodes**	71 (70)	104 (103)
**Edges**	268	610
**Positives**	171	296
**Negatives**	97	314
**Modularity**	0.629	0.459
**Modules**	6	6
**Network diameter**	7	4
**Average degree**	7.549	11.731
**Weighted degree**	1 793	-0.258
**Clustering coefficient**	0.631	0.614

## Discussion

In this study, we investigated the mechanisms by which changes in metabolites expression induced by antimicrobiota vaccination affects tick physiology, immunity, and vector competence. Our findings reveal that immunization of mice with a live *E. coli* vaccine—a form of antimicrobiota vaccination—induces host antibodies that modulate the tick microbiome when ticks feed on these mice. This modulation leads to decreased bacterial abundance, specifically targeting Enterobacteriaceae, and results in elevated lysine levels within the ticks. Lysine upregulates defensin gene expression and reduces *B. afzelii* colonization in ticks. Our results indicate that disruptions in the tick microbiota trigger metabolite changes, which could be explored for new tick-borne disease control strategies.

### Microbiota perturbation and lysine accumulation

Building upon our previous work demonstrating that antimicrobiota vaccines can modulate the tick microbiome in a taxon-specific manner (Mateos-Hernández et al. 2020a), we hypothesized that such modulation could lead to metabolite changes within the tick. Specifically, we observed a reduction in lysine degradation pathways in the tick microbiome following *E. coli* vaccination, suggesting a potential accumulation of lysine in the tick midgut. Our metabolomic analysis confirmed significantly higher concentrations of lysine in ticks fed on immunized mice compared to controls. Targeting Enterobacteriaceae disrupts amino acid metabolism, including lysine degradation.

Studies have demonstrated that antibiotics aimed at Gram-negative bacteria, such as Enterobacteriaceae, result in a significant reduction in lysine decarboxylase activity, leading to elevated lysine levels in the gut. These findings highlight the critical role of Enterobacteriaceae in maintaining amino acid balance and illustrate how their depletion can interfere with essential metabolic processes (Mu et al. 2019).

Similarly, in plant systems, the reduction of specific bacterial communities involved in amino acid metabolism can cause an accumulation of amino acids. For instance, the suppression of rhizosphere bacteria responsible for amino acid degradation has been shown to increase amino acid levels in the soil, which subsequently affects plant nutrition and growth. This lysine accumulation is likely due to the decreased abundance of bacterial enzymes required for its degradation, a consequence of the targeted reduction of Enterobacteriaceae. These observations underscore the vital role of Enterobacteriaceae and similar bacterial communities in regulating amino acid metabolism across various ecosystems. (Pantigoso et al. 2022)

### Impact on tick physiology and immunity

Lysine expression was associated with increased tick weight and altered expression of immune-related genes. Specifically, we observed upregulation of defensin genes, particularly *DefMT6*, in ticks with elevated lysine levels. Defensins are antimicrobial peptides that play crucial roles in innate immunity by targeting microbial pathogens (Hajdušek et al. 2013, Fogaça et al. 2021). The increased expression of defensins suggests that lysine enhances the tick's immune response against pathogens. However, while ticks fed with lysine-supplemented blood exhibited faster feeding, no significant difference in tick weight was observed. This may be due to the small number of ticks that successfully completed engorgement in the control group.

### Reduction of *B. afzelii* colonization

A key finding of our study is the reduced colonization of *B. afzelii* in ticks with elevated lysine levels. The increased expression of *DefMT6*, which exhibited anti-*Borrelia* activity both *in vitro* and *in vivo*, likely contributes to this reduction. The ability of DefMT6 to decrease *Borrelia* load suggests that enhancing defensin expression could be a viable strategy for controlling tick-borne pathogens.

Our results corroborate previous observations that perturbations in the tick microbiota can affect pathogen survival and transmission (Nässel et al. 2010, Narasimhan et al. 2014). By demonstrating that antimicrobiota vaccination leads to metabolite changes, we provide a link between microbiota modulation and decreased vector competence.

### Implications for tick-borne disease control

The identification of metabolites with the ability to induce physiological changes in ticks opens new avenues for controlling tick-borne diseases. Targeting the tick microbiota could modify the metabolome, enhance the tick's innate immune response, and reduce pathogen transmission. Our immunization experiments significantly decreased *Borrelia* burden, highlighting the potential of this approach. Moreover, the specificity of antimicrobiota vaccines in modulating the tick microbiome without significantly affecting the host's gut microbiota underscores their safety and applicability (Wu-Chuang et al. 2023). The development of vaccines that induce host antibodies targeting key components of the tick microbiome could be integrated into existing strategies for tick control and disease prevention.

## Conclusion

Our study reveals a novel mechanism whereby microbiota perturbations—triggered by host-derived antibodies—lead to metabolite changes in ticks, ultimately influencing their physiology, immunity, and vector competence. Antimicrobiota vaccination induces lysine accumulation in ticks leading to enhanced defensin expression and reduced *Borrelia* colonization. These findings contribute to a deeper understanding of tick biology and suggest that manipulating the tick microbiome could be an effective strategy for controlling tick-borne diseases.

## Supplementary Material

fiaf082_Supplemental_Files

## Data Availability

All the datasets shown in the present study can be found at the SRA repository https://www.ncbi.nlm.nih.gov/sra (Accession numbers: PJRNA1193515).
