# Supplementary figures and images for: Antimicrobiota vaccine induces lysine-mediated modulation of tick immunity affecting Borrelia colonization
Source: FEMS Microbiol Ecol. 2025 Aug 14;101(9):fiaf082. doi: 10.1093/femsec/fiaf082 (PMC12374725; doi:10.1093/femsec/fiaf082)

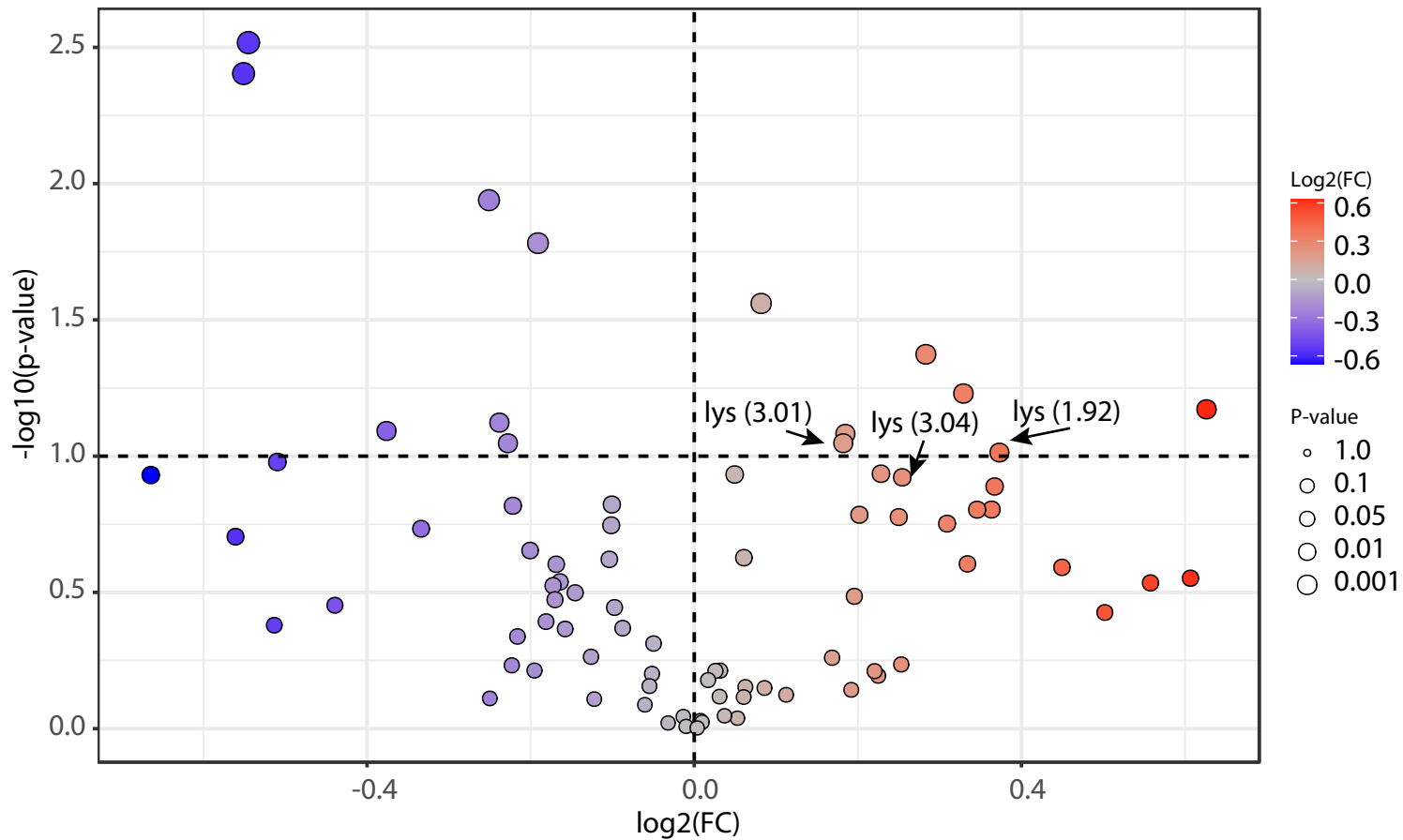

Supplement: fiaf082_Supplemental_Files [file fiaf082_supplemental_files.zip › Supplementary Figure S1.pdf]

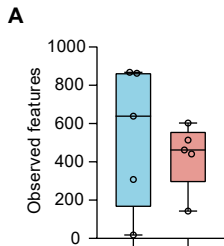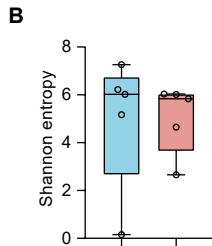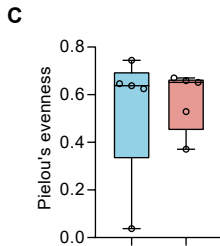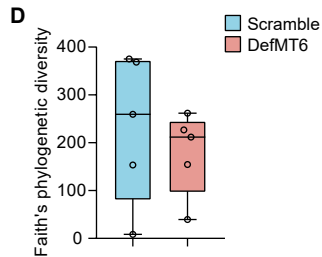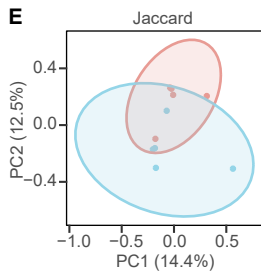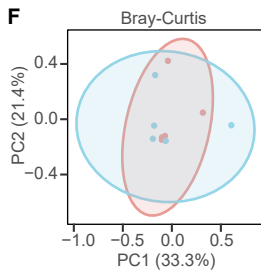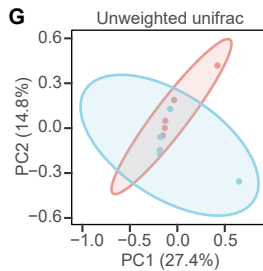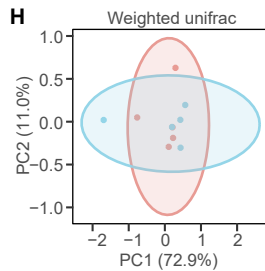

Supplement: fiaf082_Supplemental_Files [file fiaf082_supplemental_files.zip › Supplementary Figure S2.pdf]
